# Changes in urinary kidney injury molecule-1 levels after blood transfusions in preterm infants

**DOI:** 10.1038/s41598-021-91209-z

**Published:** 2021-06-03

**Authors:** Stephanie S. Turner, Jennifer M. Davidson, Mohamad T. Elabiad

**Affiliations:** 1 Ochsner Baptist Medical Center, 2700 Napoleon Ave, New Orleans, LA 70115 USA; 2 Department of Pediatrics, Division of Neonatology, College of Medicine, University of Tennessee Health Science Center, Memphis, TN 38163 USA

**Keywords:** Gastrointestinal diseases, Nephrology, Inflammation

## Abstract

Literature supports an association between transfusions and gut injury in preterm infants. We hypothesized that packed red blood (PRBC) transfusions are associated with kidney inflammation marked by a rise in urinary levels of Kidney Injury Molecule 1 (KIM-1). Prospectively, KIM-1 levels were measured before and then at 6, 12 and 24 h after a PRBC transfusion. Results are presented as mean (± SD) and median (IQR). Thirty-four infants, birth weight 865 (± 375) g, had higher pretransfusion KIM-1 levels of 2270 (830, 3250) pg/mg than what is normal for age. These were not associated with hematocrit levels. KIM-1 levels peaked between 6 and 12 h after the transfusion. Levels peaked to 3300 (1990, 6830) pg/mg; levels returned to pretransfusion levels of 2240 (1240, 3870) pg/mg by 24 h, p < 0.01. The 24-h post-transfusion KIM-1 levels were similar to pretransfusion levels, p = 0.63. PRBC transfusions in preterm infants are associated with an elevation in urinary KIM-1 levels. The mechanism of this association may be important in studying transfusion associated organ injury. KIM-1, as an inflammatory marker, may be helpful in assessing the effect of different transfusion volumes or in evaluating operational thresholds of anemia in premature infants.

## Introduction

Evidence suggests a relationship between packed red blood cell (PRBC) transfusions and organ injury. This relationship has been well documented in adults in the form of transfusion related acute lung injury (TRALI)^1^ and acute kidney injury (AKI)^2^. In a similar phenomenon in infants, transfusion related acute gut injury (TRAGI) presenting as necrotizing enterocolitis (NEC) has also been reported^3–5^. Ho et al. further evaluated this association and showed that even in the absence of overt NEC, the fecal gut inflammatory marker calprotectin does increase in a temporal manner in response to a PRBC transfusion^6^.

With evidence supporting an association between transfusions and inflammation in different body organs, it is very likely that the preterm infant neonatal kidney is similarly affected by PRBC transfusions. Studying this process has recently become easier with the availability of normal values for different kidney inflammatory markers across gestational ages^7,8^. There are several markers to choose from, but Kidney Injury Molecule 1 (KIM-1) has been shown to be a very robust molecule. KIM-1 is a proximal tubule transmembrane protein that is upregulated in post ischemic kidney injury^9^. It has the least variability across gestational ages, least variability across genders, and most stability once collected^8,10–13^.

Thus, it was hypothesized that in preterm infants, blood transfusions are associated with kidney inflammation as evidenced by an increase in urinary levels of KIM-1. Our primary objective was to evaluate the association between PRBC transfusions and KIM-1. Our secondary objective was to elucidate a timeline of when the inflammation peaks and resolves.

## Methods

### Patients

This prospective cohort study was carried out at the neonatal intensive care units of Regional One Health and LeBonheur Children’s Hospital in Memphis, Tennessee. Study period was April 1, 2016 to April 1, 2017. The study protocol was approved by and all experiments were performed in accordance with the guidelines and regulations of the Institutional Review Board at the University of Tennessee Health Science Center. Infants with a gestational age ≤ 33 weeks were enrolled upon obtaining informed consent from a parent and/or a legal guardian. Exclusion criteria included infants who had known renal anomalies (i.e. Multicystic Dysplastic Kidney, Hydronephrosis, etc.), as well as those who were noted to have ≥ stage 1 acute kidney injury (AKI)^14^ at the time of a PRBC transfusion. A resolved episode of AKI was acceptable for sample collection.

### Transfusion practices

There were no written guidelines on transfusion hematocrit thresholds during the study period. The decisions to transfuse and the volume of blood to use were at the sole discretion of the care team. Transfusions were given over 2 or 3 h. Diuretic use after blood transfusions was not routine. Furosemide was ordered based on the discretion of the primary care team. The default PRBC unit used was O negative, irradiated, leucocyte depleted and cytomegalovirus negative. The preservative used was citrate–phosphate-dextrose-adenine-1. Patients were not assigned to receive blood from a dedicated unit. Instead, one or two active O negative PRBC units were used as the source for all PRBC transfusions in the NICU until the units were exhausted or the units reached 21 days. These units were then replaced with new fresh units. Feeds were not held during transfusions.

### Urine collection and preparation

When an infant enrolled in the study had a blood transfusion ordered, a cotton ball was placed in the infant’s diaper for collection of urine creatinine and KIM-1 levels. As there was no prior data on the release dynamics of KIM-1 in urine post an insult, urine was collected before transfusion and then at 6, 12, and 24 h post-transfusion. These time points were felt to be spread out enough to detect post transfusion changes. Collections were discontinued at 24 h based on the study findings by Pitzele et al. who showed that superior mesenteric artery blood flow was blunted after a transfusion followed by a return to pretransfusion levels by 24 h post transfusion^15^. Urine was extracted from the cotton ball by placing the cotton ball into a 10 ml syringe and using the syringe plunger to squeeze the urine out into a collection tube. Urine samples were then briefly stored in a refrigerator before being centrifuged at 2000×*g* for 4 min to remove any sediment or cotton debris. The urine samples were then aliquoted into smaller volumes and frozen at − 20 °C. Samples were batched and analyzed for creatinine and KIM-1 levels within a 2–8 week period from time of collection. One hundredµL of KIM-1 was measured using a commercial ELISA kit (RayBiotech, Inc., Norcoss, GA). The limit of detection was 2–3000 pg/ml. To avoid dilutional effects, KIM-1 levels were standardized to urine creatinine and presented as pg KIM-1 per mg creatinine (pg/mg). The highest of the 6- and 12-h collections was designated as the peak response to the PRBC transfusion. Some samples could not be analyzed due to the contamination of the cotton ball with stool. These samples constituted missed collections.

### Data collection

Data extracted from the infant’s chart included demographic, pre-transfusion hematocrit of the patient, and the volume of PRBC transfused. In addition, the age of the transfused blood in bank days was also recorded. Clinical variables that may be associated with kidney inflammatory changes were also collected. These included respiratory support, exposure to antibiotics in the 72 h leading to the transfusion, exposure to a blood transfusion or to diuretics in the 48 h leading to a transfusion, and the volume of blood given during the transfusion. Blood pressures (systolic, mean, and diastolic) were documented within 2 h from the time hematocrit was collected. Hypotension was defined as any result below the 95% confidence limit for age^16^. A positive blood culture was used to confirm true sepsis. In intubated infants, the respiratory severity score (RSS) was calculated as the product between mean airway pressure and FiO_2_.

### Sample size calculation

There are no previous studies that used KIM-1 levels to characterize the magnitude of kidney inflammation associated with PRBC transfusions. Using the normal level reported for an infant at 26 weeks gestation of 918 pg KIM-1/mg UCr, we used a standard deviation of 1000 pg KIM-1/mg UCr and an effect level of 1000 pg KIM-1/mg UCr change in urine levels to calculate the sample size^7^. Based on this, a group of 32 transfusions should help achieve a statistical power of 0.8 and an alpha error at 0.05.

### Statistics

SAS 9.4 (SAS Institute Inc., Cary, NC) was used for the statistical analyses. Shapiro–Wilk test was used to test for normality. An independent samples t-test was used to compare normally distributed groups, and the Mann–Whitney U test was used when the groups where not normally distributed. KIM-1 levels where not normally distributed; a natural log was used to normalize the data across the different time points. Linear regression was used to test the association between different continuous independent variables on baseline and peak levels of KIM-1. Comparisons between changes in normalized KIM-1 levels over time were carried out using repeated measures ANOVA. A chi- square test was used to describe the association between different categorical variables. When the number of cells was less than five, a Fisher’s exact test was used. All tests were two-tailed; p < 0.05 was considered as statistically significant. Data were presented as means (± standard deviation) and as medians with first and third quartiles.

## Results

Thirty-four preterm infants were enrolled in the study, Table 1. Simultaneous results were available on pretransfusion and peak KIM-1 levels from 32 infants and on all three time points from 29 infants. At the time of sample collections, 14 infants were intubated, 15 had received diuretics, 2 had received a blood transfusion in the 48 h prior, 12 infants had received antibiotics in the immediate previous 72 h, and none had a positive blood culture. The antibiotics used were amikacin, cefazolin, ceftazidime, clindamycin, gentamicin, and/ or vancomycin. Nine infants were on a combination of vancomycin and amikacin or gentamicin. Of these 9 infants, 4 had drug levels that were high and needed medication dose adjustments. There was no association between pretransfusion degree of anemia based on hematocrit levels and pretransfusion KIM-1 levels. Other clinically important variables also did not show any association with pretransfusion KIM-1 levels, Table 1.

**Table 1:** Simple linear regression evaluating the association between variables with pretransfusion or peak KIM-1 levels.

Variable	Association with pretransfusion KIM-1 Levels		Association with peak KIM-1 Levels	
	b coefficient	p value	b coefficient	p value
Gestational age, 25 (24, 28) weeks	− 0.01	0.83	− 0.01	0.88
Gender, male n = 15	− 0.62	0.09	− 0.48	0.12
Race, Black n = 25	0.43	0.3	0.42	0.21
Birth weight, 865 (± 375) g	0.01	0.3	0.01	0.96
PMA at transfusion, 31 (± 5) weeks	− 0.04	0.36	0.02	0.57
Age at transfusion, 29 (20, 47) days	− 0.01	0.32	0.01	0.2
Serum creatinine, 0.4 (0.4, 0.5) mg/dl	− 0.52	0.49	− 0.43	0.48
Prior history of acute kidney injury, n = 4	− 0.6	0.3	− 0.49	0.29
Transfusions in preceding 48 h, n = 2	0.21	0.79	0.24	0.71
Pretransfusion KIM-1, 2270 (830, 3250) pg/mg	1	< 0.01	0.36	< 0.01
FiO_2_, 30 (21, 40) %	0.01	0.33	0.01	0.53
Respiratory severity score, 4.1 (3.3, 6.8)	0.05	0.66	0.01	0.9
Intubated, n = 14	0.08	0.83	0.13	0.67
Antibiotics in preceding 72 h, n = 12	− 0.03	0.94	0.2	0.54
Diuretics in preceding 48 h, n = 15	− 0.04	0.91	0.1	0.76
Hematocrit at transfusion, 27 (± 4) %	0.05	0.36	0.04	0.42
Volume of transfusion, ml/kg	0.17	0.03	− 0.05	0.46

*PMA* post menstrual age.

Results were presented as mean (± SD) or median (IQR).

**Figure 1:**
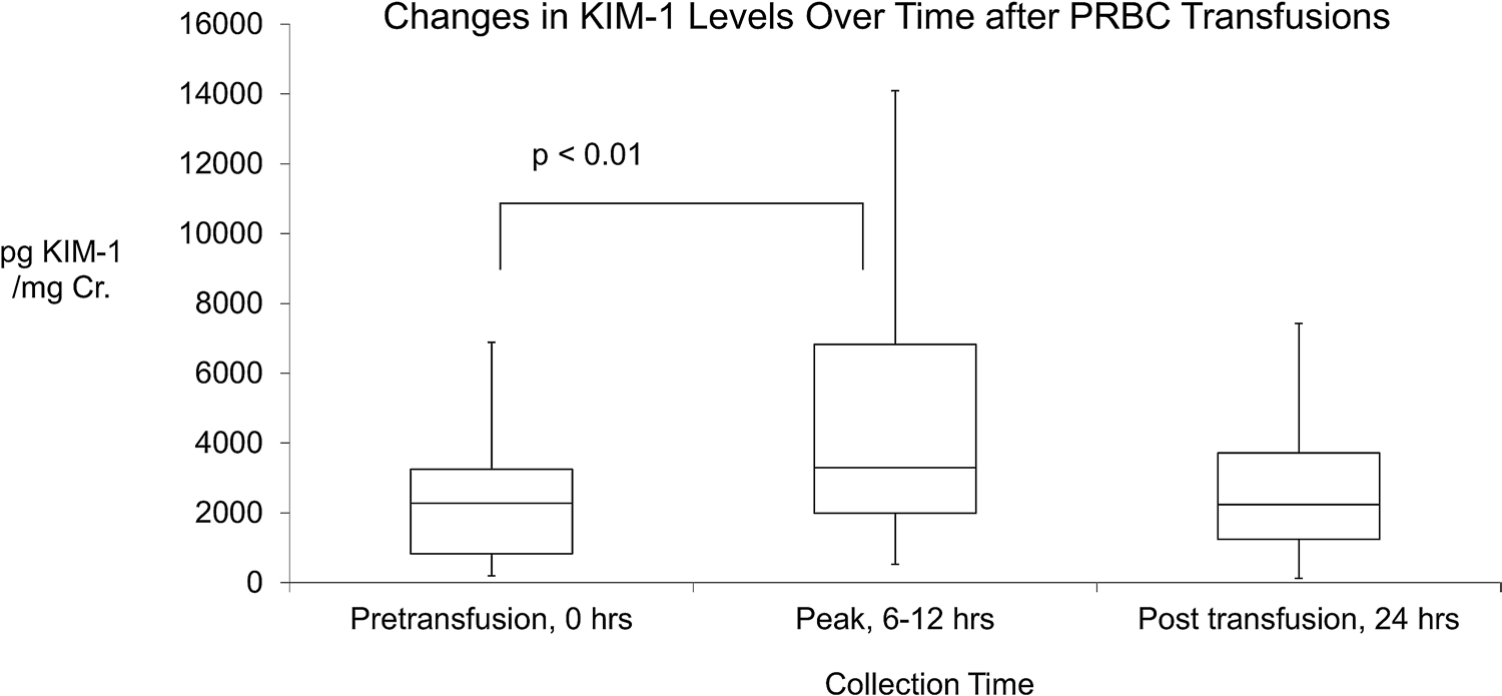
Changes in KIM-1 Levels with PRBC transfusions: KIM-1 levels were significantly higher 6 to 12 h after the transfusion but by 24 h had no significant difference from pretransfusion levels.

Blood transfusions were associated with significant changes in the natural log KIM-1 levels over the three time points, p < 0.01. The median pre-transfusion KIM-1 levels increased from 2270 (830, 3250) pg/mg and peaked at 3300 (1990, 6830) pg/mg. At 24-h post-transfusion KIM-1 levels dropped to 2240 (1240, 3870) pg/mg, not significantly different from the pre-transfusion levels, p = 0.63, Fig. 1. In multivariable analyses adjusted for pretransfusion KIM-1 levels and volume of blood received, blood transfusions were still associated with significant changes in KIM-1 levels over the three time points, p < 0.01. Excluding the four infants with potentially nephrotoxic levels from the analysis did not change the main result. Blood transfusions were still associated with significant changes in the natural log KIM-1 levels over the three time points, p < 0.01.

All 12 infants that received a 10 ml/kg blood transfusion had a higher peak KIM-1 levels when compared to pretransfusion levels. However, only 12 of 20 15 ml/kg transfusions were associated with an increase in KIM-1 levels from baseline, p = 0.014, Table 2.

**Table 2:** Differences in variables between infants that received 10 ml/kg vs. 15 ml/kg blood transfusion volumes.

Variable	10 ml /kg volume	15 ml /kg volume	p value
Number of infants	12	22	0.33
Gestational age, weeks	25 (24, 29)	25 (24, 28)	0.51
Gender, male, n (%)	6 (50)	9 (41)	0.61
Race, Black = 40	8 (67)	17 (77)	0.5
Birth weight, g	795 (630, 1055)	815 (570, 990)	0.79
PMA at transfusion, weeks	33 (± 6)	30(± 4)	0.06
Age at transfusion, days	29 (24, 64)	26 (19, 45)	0.29
Serum creatinine mg/dl	0.45 (0.35, 0.50)	0.40 (0.40, 0.50)	0.81
Prior history of acute kidney injury	2 (17)	2 (9)	1
Transfusions in preceding 48 h	1 (8)	1 (5)	0.47
Pretransfusion KIM-1 pg/mg	1032 (625, 2365)	2610 (1560, 5080)	0.04
FiO_2_, %	28 (21, 36)	30 (23, 45)	0.43
Respiratory severity score	3.9 (3.9, 4.3)	4.3 (3.3, 6.8)	0.95
Intubated	5 (42)	9 (41)	0.97
Antibiotics in preceding 72 h, n = 21	4 (33)	8 (36)	1
Diuretics in preceding 48 h, n = 27	4 (33)	11 (50)	0.48
Hematocrit at transfusion (%)	26 (± 4)	27(± 4)	0.35
Storage, 15 (12, 18) days	16(± 4)	14(± 4)	0.27
Systolic blood pressure	71 (55, 77)	69 (62, 74)	0.96
Mean blood pressure	53 (37, 55)	50 (44, 54)	1.0
Diastolic blood pressure	32 (26, 46)	38 (33, 43)	0.60
Number of transfusions with positive increase	12 (100)	12 (60)	0.014

Results were presented as mean (± SD) or median (IQR).

*PMA* post menstrual age.

Infants that received a 10 ml/kg volume of PRBC transfusion had a significantly lower pretransfusion KIM-1 level. They also had a trend of having a higher post menstrual age (PMA) at the time of the transfusion, Table 2. Adjusting for PMA at time of transfusion showed no difference between the 10 ml/kg vs the 15 ml/kg groups in their pretransfusion KIM-1 levels, p = 0.28. One infant in the group that received 15 ml/kg blood transfusion volume had a diastolic blood pressure less than the 95% C.L. However, there were no statistical differences between the two group in pretransfusion blood pressure indices, Table 2.

In 63% of the time, infants developed a peak in their post transfusion KIM-1 levels by 6 h after the conclusion of the blood transfusion. However, there was no significant differences in pretransfusion KIM-1 levels between those infants who peaked at 6 h 2270 (1040, 3040) pg/mg and those who peaked at 12 h 2740 (830, 4450), p = 0.69. One infant developed NEC 24 h after receiving a blood transfusion. His pretransfusion level was 790 pg/mg, peaked by 6 h at 980 pg/mg, and dropped to 180 pg/mg by 12 h post transfusion. At 24 h, the infant had clinically overt NEC and his level had increased to 3070 pg/mg.

## Discussion

This study demonstrated a temporal association between the intervention of receiving a blood transfusion and the outcome of an increase in the level of the kidney inflammatory marker KIM-1. Furthermore, it suggests a timeline of when KIM-1 levels peak and how long it takes for them to return to the pretransfusion status. This timeline may help in further evaluating the pathophysiology of transfusion associated organ injury.

In infants undergoing cardiac surgery, Bojan et al. reported an association between blood transfusions and the development of AKI^17^. Uygur et al.^18^ also reported on infants undergoing cardiac surgery and found that postoperative KIM-1 levels were higher than preoperative levels. However, blood transfusions were not mentioned in their evaluation. In non-surgical infants, the evidence is scarce about an association between blood transfusions and kidney injury or inflammation. There is however extensive evidence for gut injury and on the different mechanisms of how blood transfusions may be involved^19,20^. Anemia and storage time are two common important factors.

Anemia, both chronic and acute has been proposed to explain the mechanism for TRAGI^21,22^. As a premature infant gets older, it is common practice to accept lower hematocrit levels before giving a PRBC transfusion^23^. This was no different in our study results. This accepted norm of anemia is supported by evidence from randomized studies that showed no difference in morbidities between restrictive compared to liberal thresholds of hemoglobin to decide on when to give a blood transfusion^24,25^. Conversely, when hemoglobin thresholds were maintained at higher levels, exposure to blood transfusions was protective against TRAGI^26^. Our study did not show any association between severity of anemia and pretransfusion KIM-1 levels. This may be due to a small sample size. Another factor could be the degree of anemia and any potential resulting ischemia. Severe anemia may have led to higher pretransfusion levels across the study population resulting in loss of any significant association between pretransfusion KIM-1 levels and hematocrit. This is supported by the fact that our pretransfusion KIM-1 levels were higher than normal levels proposed for similar gestational age infants^7^, 2270 (830, 3250) pg/mg compared to 637 (416, 976) pg/mg respectively at 30 weeks gestational age^7^. To properly evaluate the association between KIM-1 and anemia, KIM-1 levels would need to be measured on non-anemic infants to compare their KIM-1 levels with anemic infants just before they receive a transfusion.

KIM-1 levels were lower in infants who received 10 ml/kg PRBC as compared to those who received 15 ml/kg. It is possible that the care providers may have chosen a lower volume of transfusion for infants that looked less ill. After adjusting for PMA at the time of transfusion, there was no difference between the two groups of blood transfusion volumes and their pretransfusion KIM-1 levels. There was also no difference in the peak KIM-1 levels between the two groups. Infants that received 10 ml/kg transfusion volume were more likely to have a positive change in their KIM-1 level from baseline. Again, this association weakened and was no longer significant after adjusting for PMA at the time of the transfusion. Future studies may need to evaluate the volume of the transfused blood as a contributing factor in the pathophysiology of transfusion associated organ injury.

The age of stored blood is another factor proposed to explain transfusion associated organ injury. In our study, we did not find such an association. One explanation could be that all transfusions were received at ≤ 21 PRBC bank days, an age after which longer storage time has been associated with TRAGI^6^. Nevertheless, the pathophysiology proposed to explain TRAGI due to longer storage time may explain the behavior in serum KIM-1 levels in the 24 h post transfusion. It has been shown that processing RBCs for storage decreases their nitric oxide (NO) content. This in turn affects the RBCs ability to cause regional vasodilation^27^. With longer storage time, RBCs develop a significant disruption in their cell morphology^28^, and this in turn leads to RBC breakdown. Released cell free hemoglobin scavenges NO and consequently limits its availability for tissue vasodilation^29^. This “storage lesion” translates into a lower ability to improve regional tissue oxygenation in the immediate period after a blood transfusion. Interestingly, it has also been shown that stored blood may be renitrosylated thus regaining its vasodilation abilities. In an in vivo canine model, coronary artery blood flow was significantly greater with infusion of renitrosylated RBC as compared to S-nitrosothiol depleted RBC^27^. Based on these findings, it is possible that several hours after transfusion, the infused RBCs get renitrosylated and regain their vasodilation abilities. This may explain why in the immediate post transfusion period, the intestinal tissue perfusion is decreased, and in the follow up period, when RBCs are renitrosylated, intestinal perfusion is back to baseline. The increase in KIM-1 levels 6–12 h post transfusion and their return to baseline line mirrors the timeline Pitzele et al. observed when evaluating the time needed for the return of superior mesenteric blood flow velocities to pretransfusion levels^15^.

Our study had some limitations. We were not able to evaluate for an association between blood transfusions and the development of AKI as the study design limited our ability to collect serum creatinine in the immediate post transfusion period. The study was also not powered to evaluate for differences in transfusion volumes and their effect on peak KIM-1 levels. We also were not able to show any association between pretransfusion hematocrit levels in relation to KIM-1 levels. We speculate that normal KIM-1 levels reflect a status of wellbeing whereby hematocrit levels are meeting the metabolic oxygen needs of the body. If true, this information would be very helpful to improve transfusion hematocrit thresholds based on individual infant needs. Another limitation was the small number of time points collected to evaluate the response of KIM-1 to transfusions. By limiting the last collection to 24 h post transfusion, it was not possible to determine if further time points would show whether an amelioration of the anemia would be associated with a significant drop in KIM-1 levels when compared to pretransfusion levels. There were a few hemodynamic limitations. First, there were no infant controls in this study. This would be important to evaluate how KIM-1 is excreted in non-anemic infants. Second, the availability of other important hemodynamic information like the ductus arteriosus status would have clarified any associations between hemodynamic instabilities and changes in KIM-1 levels. Samples were not stored at − 80 °C but at − 20 °C and up to two months. As each patient’s urine samples were collected and stored together, it is unlikely that there was selective sample deterioration on some time point samples but not on others. Conversely, one of the major strengths of using KIM-1 in the study is its rapid response time and its non-invasive means of collection.

## Conclusion

In summary, we conclude that PRBC transfusions in infants are associated with a transient, yet subtle, inflammatory status as evidenced by elevated KIM-1 levels in the urine following PRBC transfusion. Future studies may elect to study if blood transfusions negatively influence renal artery perfusion. KIM-1 as an inflammatory marker may be considered in future studies evaluating the responses of the body to different transfusion volumes. KIM-1 may also help in studies that evaluate and assist with understanding the operational thresholds of anemia in premature infants.

## Data Availability

The datasets generated during and/or analyzed during the current study are available from the corresponding author on reasonable request.
